# A systematic review of studies evaluating Australian indigenous community development projects: the extent of community participation, their methodological quality and their outcomes

**DOI:** 10.1186/s12889-015-2514-7

**Published:** 2015-11-21

**Authors:** Mieke Snijder, Anthony Shakeshaft, Annemarie Wagemakers, Anne Stephens, Bianca Calabria

**Affiliations:** National Drug and Alcohol Research Centre (NDARC), UNSW, Randwick, NSW 2052 Sydney, Australia; Public Health and Society, Department of Social Sciences, Wageningen University and Research Centre (WUR), Wageningen, The Netherlands; The Northern Research Futures Collaborative Research Network, The Cairns Institute, James Cook University (JCU), Cairns, Australia; National Centre for Epidemiology and Population Health, Research School of Population Health, College of Medicine, Biology and Environment, Australian National University, Canberra, Australia

**Keywords:** Community development, Indigenous, Aboriginal, Torres Strait Islander, Health promotion, Community participation, Empowerment, Methodological quality, Qualitative, Quantitative

## Abstract

**Background:**

Community development is a health promotion approach identified as having great potential to improve Indigenous health, because of its potential for extensive community participation. There has been no systematic examination of the extent of community participation in community development projects and little analysis of their effectiveness. This systematic review aims to identify the extent of community participation in community development projects implemented in Australian Indigenous communities, critically appraise the qualitative and quantitative methods used in their evaluation, and summarise their outcomes.

**Methods:**

Ten electronic peer-reviewed databases and two electronic grey literature databases were searched for relevant studies published between 1990 and 2015. The level of community participation and the methodological quality of the qualitative and quantitative components of the studies were assessed against standardised criteria.

**Results:**

Thirty one evaluation studies of community development projects were identified. Community participation varied between different phases of project development, generally high during project implementation, but low during the evaluation phase. For the majority of studies, methodological quality was low and the methods were poorly described. Although positive qualitative or quantitative outcomes were reported in all studies, only two studies reported statistically significant outcomes.

**Discussion:**

Partnerships between researchers, community members and service providers have great potential to improve methodological quality and community participation when research skills and community knowledge are integrated to design, implement and evaluate community development projects.

**Conclusion:**

The methodological quality of studies evaluating Australian Indigenous community development projects is currently too weak to confidently determine the cost-effectiveness of community development projects in improving the health and wellbeing of Indigenous Australians. Higher quality studies evaluating community development projects would strengthen the evidence base.

## Background

The health gap between Indigenous and non-Indigenous Australians has been well documented [1–3]. Systematic literature reviews, however, have consistently concluded that evaluations of interventions aimed at reducing this health gap lack methodological rigour [4–12]. In addition to improving the methodological quality of the evidence-base, the need for greater community participation in, and control of, Indigenous health promotion research have been advocated [13–15].

Community participation has long been argued as being an essential factor in successful health promotion initiatives [16–18]. A recent meta-analysis concluded that community participation is effective when used in health promotion projects because it engenders greater community motivation and increases the sustainability of projects [19]. Although the review did not include Indigenous communities, the principle of community participation is highly relevant to Indigenous Australians and has great potential to improve Indigenous health. The history of dispossession and disempowerment experienced by Indigenous people highlights the importance of the full and active participation of community members to develop plausible solutions to the problems they themselves have identified [9, 20–25]. The community development approach strives to empower Indigenous communities to develop and utilise skills that will enable them to more directly address the risk factors that determine their health status [26].

Despite the potential of community development approaches for improving Indigenous health outcomes, there has been no systematic examination of the extent to which they have engendered community participation and little analysis of their effectiveness. The only existing systematic review of Indigenous community development, published in 2007, evaluated 17 projects implemented in Indigenous communities in Australia, Canada, New Zealand and the United States [9]. This review emphasised that high levels of community participation were a critical factor in the success with which community development projects were implemented, however, it did not assess the level of community participation nor systematically assessed the methodological qualities of the studies. This lack of project evaluation has made it difficult to confidently estimate the extent to which community development projects have improved the health and life expectancy of Indigenous People.

This systematic review aims to identify the extent of community participation in community development projects implemented in Australian Indigenous communities, critically appraise the qualitative and quantitative methods used in their evaluation, and summarise their outcomes.

## Methods

### Identification of publications

**Fig. 1:**
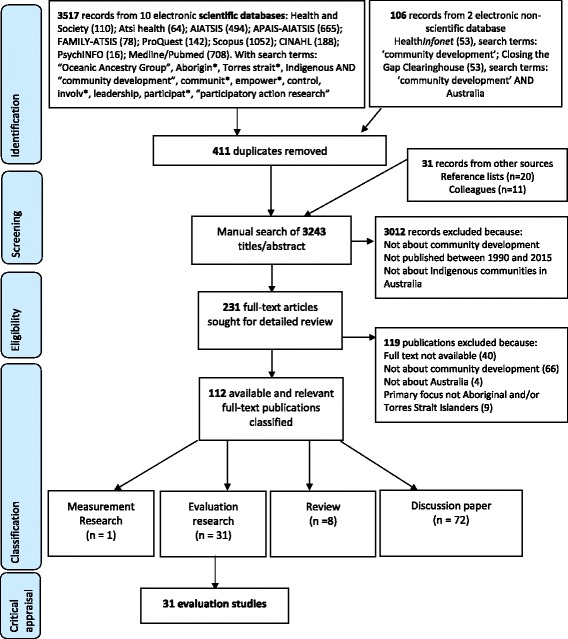
PRISMA flow diagram: systematic search identifying evaluation studies of Australian Indigenous Community Development projects

The peer-reviewed and grey literature were searched to identify studies evaluating Indigenous community development projects in Australia, published between 1990 and 2015. Twenty-five years of community development projects was judged to be sufficient to provide an overview of the most recent projects. Figure 1 summarises the databases searched, the search terms used, the eligibility criteria and the classification process based on the PRISMA flow diagram [27].

Ten peer-reviewed databases were searched: Health and Society, Aboriginal and Torres Strait Islander Health Bibliography (ATSIHealth), AIATSIS, APAIS-AIATSIS, FAMILY-ATSIS, ProQuest, Scopus, CINAHL, PsychINFO, and Medline/Pubmed. Two grey literature databases were searched: Health*Infonet* and Closing the Gap Clearinghouse. The electronic database search identified 3623 publications and 411 duplicates were removed. Reference lists of the identified publications were scanned which identified an additional 20 publications. Another 11 publications were received from researchers in the field. The resulting 3243 publications were organised in Reference Manager Endnote [28].

### Screening and eligibility

The titles and abstracts of the identified 3243 publications were read to determine their eligibility for inclusion against three criteria: 1) relevance to a community development project, including: projects focusing on community ownership, empowerment, local leadership and decision making, adopting a long-term strategy, having a focus on sustainability or having a bottom up approach (i.e. starting from the community) [29, 30]; 2) published between 1990 and 2015; and 3) a primary focus on Indigenous communities in Australia. A total of 231 publications met all three criteria. The full text versions of these 231 publications were sought for detailed review, of which 112 were available and relevant to this review.

### Classification

One hundred and twelve publications were classified into four categories derived from previous research reviewing Indigenous health initiatives [4, 10, 31], defined as follows. *Measurement research:* the development, testing or evaluation of measurement tools. *Evaluation research:* an evaluation of an Indigenous community development project or policy. *Review:* including summaries, critical or systematic reviews and/or meta-analysis; and *Discussion paper:* general discussion of Indigenous community development. Thirty one publications identified as studies evaluating community development projects in Indigenous Australian communities were critically appraised.

### Extent of community participation

Replicating previous analyses [32, 33], the extent of community participation was assessed using Pretty’s participation typology, which describes seven levels of community participation ranging from no participation to self-mobilisation (i.e. completely top-down to completely bottom-up) [32, 34]. Given community participation can vary during the lifetime  of a project, the extent of participation was assessed separately for four phases of project development: diagnosis (identifying a community’s priorities); development (of appropriate strategies to address the priorities); implementation (of the strategies); and evaluation (of the effectiveness of the project) [32, 35, 36]. The level of community participation in the 31 studies were assigned a score between 1 and 7 for each phase of project development. Detailed descriptions of different levels of community participation in relation to scores 1 to 7, and with respect to all four phases, are provided in Table 1 and are summarised as follows: no participation (score 1); passive participation (score 2 – the community was only informed about the project); participation by information (score 3 – information was collected from the community without their participation and without providing feedback); participation by consultation (score 4 – information was collected from the community, feedback was given and further inclusion of community was sought); functional participation (score 5 – community collaboration, but on outsiders’ terms); interactive participation (score 6 – collaboration on mutually defined terms); and self-mobilisation (score 7 – outsider’s work in community on community’s terms). This scoring was independently conducted by two of the authors (MS and AW), which resulted in agreement for 22 studies (71 %). The nine studies on which authors disagreed were blindly reviewed by a third author (ASt). For four studies, the score allocated by the third reviewer was the same as the score allocated by one of the first two reviewers and so that score was used, increasing the agreement rate to 84 % for 26 of the 31 studies. For the five studies where there was no agreement, the scores allocated by the first author (MS) were used.

**Table 1:** Definitions of the seven levels of community participation in the four phases of project development

Seven levels of community participation	Four phases of project development			
	Diagnosis	Development	Implementation	Evaluation
1. No participation	Completely top-down, community is not informed about or asked about issues in their community.	Top-down, community is not informed about the development of the project.	Top-down, community is not informed about the implementation of the project, only about activities they’re involved in	Top-down, community receives no information about evaluation.
2. Passive participation	Outsiders decide on the issues that need to be addressed, community is informed.	Outsiders control development, community is informed, but has no input.	Outsiders control the implementation, community is informed, but has no input.	Outsiders control the evaluation, community is informed, but has no input.
3. Participation by information	Outsiders have control, community participates by providing information about their community. No feedback to the community and no checking for agreements.	Outsiders have control over development, community potentially provides information about what they want, but outsiders don’t necessarily respond to this.	Outsiders control implementation, community might provide information useful for implementation, but outsiders don’t necessarily listed to this.	Outsiders control evaluation, community provides information through surveys and/or interviews, focus groups. Findings are not shared or checked for accuracy.
4. Participation by consultation	Outsiders define problems and consult with community about their agreement, using outsider defined processes.	Outsiders consult with community about potential projects to develop, but outsiders make final decision.	Community participates in activities decided upon by the outsiders	Outsiders define evaluation process, community provides information and might make suggestions for improvement and feedback provided
5. Functional participation	Outsiders have predetermined goals and community assists in defining issues within those goals, outsiders make final decisions.	Community works together with outsiders to develop projects decided upon by the outsiders.	Community and outsiders work towards implementation of projects, based on outsiders’ goals and processes.	Community and outsiders work together in evaluation, based on goals as set by the outsiders.
6. Interactive participation	Outsiders and community work together to identify the issues in the community and set goals for the project.	Outsiders and community work together to develop suitable projects to address the agreed upon goals.	Community and outsiders implement the developed projects together, community has control and uses local resources.	Evaluation methods are decided upon together and conducted in partnership.
7. Self-mobilisation	Completely bottom-up, community identifies their own issues and sets their own goals, might contact outsiders to assist them where needed.	Bottom-up, community makes decisions about project development, apply for funding and potentially contact outsiders where needed	Community implements projects, contacts outsiders for resources where needed, but remains in control over resources.	Community conducts evaluations, potentially contacts outsiders for assistance, but stays in control over evaluation.

Source: adapted from Pretty (1995) and Wagemakers et al. (2008)

### Critical appraisal of methodology

#### Qualitative methods

The methodological quality of the qualitative study components was assessed by adapting Long and Godfrey’s qualitative study evaluation tool, which was developed to appraise evaluations of health and social care interventions [37]. This tool has 4 sections: 1) phenomenon studied and context; 2) ethics; 3) data collection, analysis and potential research bias; and 4) policy and practical implications. The latter two were used in this review because they relate specifically to evaluation issues. Data collection includes the need for clear descriptions of the data collection process (e.g. recruitment strategies, data collection procedures, specifying the interview questions, methods of recording data and the extent to which the data collection process was tailored to specific communities). Data analysis includes the description of the data analysis, the provision of adequate evidence to support the analysis (including data extracts, triangulations and descriptions of reliability) and whether the findings are interpreted in line with existing theories and literature. Potential researchers’ bias assesses whether the position of the researcher is outlined in the study and its potential influence on the data collection and analysis. The policy and practical implications include an analysis of the populations and settings to which the findings are generalisable, the implications for policy or practice, and the extent to which the methods justify the conclusions.

#### Quantitative methods

The methodological quality of the quantitative study components was appraised using the Dictionary for Effective Public Health Practice Project Quality Assessment tool for Quantitative studies [38], which was developed to review public health studies and has been used in other systematic reviews in the Australian Indigenous health field [4, 10]. Sections A-F (A - selection bias, B - study design, C - confounders, D - blinding, E - data collection methods, F - withdrawals and drop-out) are rated categorically as strong, moderate or weak. Sections G (intervention integrity) and H (analysis appropriateness) comprise summaries of the relevant information rather than categorical ratings. In addition to sections A-H, this tool advocates a summary rating defined as weak (two or more weak scores are given), moderate (1 weak score is given) or strong (no weak scores are given).

#### Scoring for critical appraisal of methodology

Scores against both the qualitative and quantitative evaluation criteria were allocated by author MS. A random selection of 25 % of studies were assessed by a blinded coder (ASt). There was agreement for 70 % of these studies. Disagreements were resolved in consultation between the two coders.

### Outcomes of the studies

The outcomes of the studies evaluating Indigenous community development projects are summarised.

## Results

Thirty-one studies evaluating Indigenous community development projects in Australia were identified. Ten (32 %) were published in the peer reviewed literature [39–48].

### Extent of community participation

Table 2 summarises the level of community participation across the four phases of project development for each study. Table 3 summarises the number of studies relevant to each of the seven levels of community participation, separately for the four phases of project development. The highest levels of participation (level 5 to level 7), were found in the Diagnosis phase for ten studies (32 %) [39, 43, 44, 48–54], in the Development phase for 13 studies (42 %) [39–44, 46, 49–52, 54, 55], in the Implementation phase for 17 studies (55 %) [39–44, 46, 48–54, 56–58] and in the Evaluation phase for 7 studies (22 %) [39, 40, 43, 44, 50, 53, 59]. Four studies (13 %) had at least level 5 participation in all phases of the project [39, 43, 44, 50]. The participation of the community was described with insufficient detail to be assessed (unknown category) for ten studies in the Diagnosis phase (32 %) [42, 45, 57, 58, 60–65], seven in the Development phase (23 %) [47, 48, 57, 60, 61, 64, 66], four (12.9 %) in the Implementation phase [47, 61, 63, 66] and two (7 %) in the Evaluation phase [46, 67].

**Table 2:** Level of community participation in each phase of project development for each study

First author (year)	Four phases of project development			
	Diagnosis	Development	Implementation	Evaluation
Gauld et al. (2011) [40]	1^a^	2–5^b^	2–5	5
Green et al. (2009) [67]	4	4	4	UNK
McMurray (2012) [49]	7	6	6	3
Parker et al. (2006) [48]	6	UNK	5	4
Murphy et al. (2004) [41]	3	7	5	4
Hunt (2010a) [59]	7	7	7	6
CLC (2012a) [60]	UNK	UNK	4	3
CLC (2012b) [51]	7	7	7	3/4^c^
CLC (2012c) [61]	UNK	UNK	UNK	3/4
CLC (2012d) [62]	UNK	3	3	4
CLC (2012e) [63]	UNK	4	UNK	3
Taylor (2005a) [56]	1	UNK	5	4
Taylor (2005b) [64]	UNK	UNK	4	4
Ramsay (2005a) [57]	UNK	UNK	5	4
Ramsay (2005b) [68]	1	3	4	4
Burchill (2005) [65]	UNK	3	4	4
Higgins (2005) [52]	7	7	7	4
Bromfield (2005) [55]	1	5	4	4
Ramsay (2005c) [66]	2	UNK	UNK	4
Tsey (2003) [69]	1	3	4	3
Tsey et al. (2004); [39]	5	6	6	5
Smith (2004) [53]	7	3	6	6
Lee et al. (2008) [42]	UNK	2–5	4–5	4
Tyrrell et al. (2003) [43]	6	6	6	5
Guenther (2011) [58]	UNK	2	5	2
Salisbury (1998) [44]	5	5/6	5/6	6
Hunt (2010b) [59]	1	2	4	5
Moran (2003/2004) [45]	UNK	4	4–1	2
McCalman (2005) [54]	7	7	7	4
Jarvie (2008) [46]	1	5	5	UNK
Shannon et al. (2001) [47]	3	UNK	UNK	3

^a^Possible scores range from 1 to 7: 1 = no participation; 2 = passive participation; 3 = participation by information; 4 = participation by consultation; 5 = functional participation; 6 = interactive participation; 7 = self-mobilisation, UNK = unknown [32, 34]

^b^Participation varied within the phase

^c^Participation was somewhere in between these levels

**Table 3:** Number of studies across the levels of community participation and phases of project development

Seven levels of community participation	Four phases of project development			
	Diagnosis	Development	Implementation	Evaluation
1. No participation	7			
2. Passive participation	1	3		2
3. Participation by information	2	3		6
4. Participation by consultation	1	5	10	14
*Least active involvement sub-total (levels 1–4)*	*11*	*11*	*10*	*22*
5. Functional participation	1	4	8	4
6. Interactive participation	3	4	5	3
7. Self-mobilisation	6	5	4	0
*Most active involvement sub-total (levels 5–7)*	*10*	*13*	*17*	*7*
Unknown	10	7	4	2
Total	31	31	31	31

*Note:* No participation = community did not participate

Passive participation = the community was only informed about the project

Participation by information = information was collected from the community without their participation and without providing feedback.  Participation by consultation = information was collected from the community, feedback was given and further inclusion of community was sought

Functional participation = community collaboration on outsider’s terms

Interactive participation = collaboration on mutually defined terms

Self-mobilisation = outsider’s work in community on community’s terms [32, 34]

### Methods used in studies

Twenty-one studies (67 %) used qualitative methods only [39–41, 48, 49, 51, 52, 55–57, 59–69], two (7 %) used quantitative methods only [46, 47], and eight (26 %) used mixed methods [42–45, 53, 54, 58, 59]. Qualitative data were collected using semi-structured interviews in 24 studies [39, 40, 42, 43, 45, 49–53, 55–58, 60–69], document analysis (*n* = 15 [42, 49, 51, 52, 55–57, 60–66, 68]), focus groups (*n* = 9 [39, 40, 43, 45, 48–50, 53, 58]), participant observation (*n* = 6 [42, 43, 53, 54, 58, 69]) and photovoice [70] (*n* = 2 [50, 53]). Quantitative data collection methods included surveys in three studies [45, 48, 58], hospital/clinical records (*n* = 4 [43, 44, 47, 53]), school records (*n* = 2 [42, 58]), police records (*n* = 1 [42]), store records (*n* = 1 [43]) and ABS census data (*n* = 1 [46]).

### Methodological quality of studies with a qualitative component

All 29 studies with a qualitative component (including mixed methods studies) provided some description of the evaluation methods used (Table 4). Twelve studies (41 %) gave detailed descriptions of the data collection process, including participant recruitment, focus group procedures and a clear description of which data were recorded [39, 41, 49–51, 53, 58, 60–63, 67]. Four of these twelve studies (14 %) provided the interview questions [51, 58, 60, 67] and one study (4 %) described in detail how the data collection methods were tailored to ensure their cultural appropriateness [49]. The data analysis methods were described in detail in seven studies (24 %) [39, 42, 50, 54, 58, 67, 69]. The potential for researcher bias was described in seven studies (24 %) [39, 44, 45, 49, 53, 58, 69]. Three studies (10 %) did not discuss the implications of their findings [52, 59, 65].

**Table 4:** Critical appraisal of qualitative components of studies evaluating Indigenous community development projects (*n* = 29)

First author (year)	Data collection	Data analysis	Potential bias	Implications
Qualitative only studies (*n* = 21)				
Gauld et al. (2011) [40]	Little detail	Not described	Not described	Generalised to far northern Queensland communities
Green et al. (2009) [67]	Detailed description, including interview questions	Detailed description and linked to literature	Not described	Generalised to organisations working with Indigenous communities in Australia and policy
McMurray (2012) [49]	Detailed description of field work	Not described	Position of researcher described	Implications for the funding agency
Parker et al. (2006) [48]	Not described	Not described	Not described	Described for health promotion work in Indigenous communities
Murphy et al. (2004) [41]	Detailed description	Not described	Not described	Appreciative inquiry methods and culture projects
CLC (2012a) [60]	Detailed description	Description of who did analysis and triangulation	Not described	Described for organisation
CLC (2012b) [51]	Detailed description	Description of who did analysis and triangulation	Not described	Described for organisation
CLC (2012c) [61]	Detailed description	Description of who did analysis and triangulation	Not described	Described for organisation
CLC (2012d) [62]	Detailed description	Description of who did analysis and triangulation	Not described	Described for organisation
CLC (2012e) [63]	Detailed description	Description of who did analysis and triangulation	Not described	Described for organisation
Taylor (2005a) [56]	Little detail	Not described	Not described	Generalised to comparable projects
Taylor (2005b) [64]	Little detail, mention of development of evaluation tool	Not described	Not described	Described for future communities wanting to implement project
Ramsay (2005a) [57]	Little detail	Not described	Not described	Generalised to Indigenous communities with comparable issues
Ramsay (2005b) [68]	Little detail	Not described	Not described	Discussed for working with Indigenous communities
Burchill (2005) [65]	Very little detail	Not described	Not described	Not described
Higgins (2005) [52]	Very little detail	Not described	Not described	Not described
Bromfield (2005) [55]	Very little detail	Not described	Not described	Discussed for practice
Ramsay (2005c) [66]	Very little detail	Not described	Not described	Discussed for practice
Tsey (2003) [69]	Detailed description	Detailed description	Position of researcher is discussed	Generalised to community development projects and practice
Tsey et al. (2004); [39]	Very detailed description	Detailed description	Position of researcher discussed	Discussed for practice, policy and researcher
Hunt (2010b) [59]	Not described	Not described	Not described	Not described
Mixed Methods Studies (*n* = 8)				
Hunt (2010a) [59]	Detailed description of fieldwork	Described	Researcher position and bias described	Described for organisation’s community development work
Smith (2004) [53]	Very detailed description	Description of who analysed data, but not methods	Position of researcher discussed	Generalised to other communities, implications for project described
Lee et al. (2008) [42]	Detailed description	Described	Not described	Generalised to communities with similar problems.
Tyrrell et al. (2003) [43]	Not described	Not described	Not described	Discussed for practice and results
Guenther (2011) [58]	Detailed description	Detailed description	Position of researcher discussed	Discussed for policy and practice
Salisbury (1998) [44]	Little detail	Not described	Researcher position discussed	Generalised to health services in Indigenous communities
Moran (2003/2004) [45]	Detailed description	Description of who analysed data, but not methods	Researcher position and bias discussed	Discussed for practice
McCalman (2005) [54]	Very little detail	Very little detail	Not described	Discussed for practice

### Methodological quality of studies with a quantitative component

The summary ratings for all ten studies with a quantitative component were classified as weak (Table 5). The likely extent of selection bias was unclear for six studies (60 %) because description of the participant and community selection procedures was absent or insufficiently detailed [42–44, 46, 47, 59]. Five studies (50 %) used a cohort design without a control group [43–45, 53, 54], one study (10 %) used a time series design [47] and the evaluation design of the remaining four studies (40 %) was unclear [42, 46, 58, 59]. No study adequately controlled for confounding variables. None of the studies used blinding procedures. Two studies (20 %) used validated outcome measures [45, 58]. No study discussed the validity or reliability of their outcome measures.

**Table 5:** Critical appraisal of quantitative component of studies evaluating Indigenous community development projects (*n* = 10)

1st author, year	Selection bias (A)	Study design (B)	Confounds (C)	Blinding (D)	Data collection methods (E)	Withdrawal & drop-outs (F)	Intervention integrity (G)	Analysis (H)	Summary rating
Mixed method studies (*n* = 8)									
Smith (2004) [53]	Moderate	Moderate	NA	Weak	Weak	Moderate	Collection of quantitative data stopped before real community action started.	Community-level allocation, individual-level analysis. No appropriate analysis of change in child growth over time.	Weak
Lee (2008) [42]^a^	Weak	Weak	NA	Moderate	Weak	Moderate	Many youth involved in the interventions, no information on consistency, other community initiatives were running simultaneously (including stricter supply controls and rewards linked to school attendance).	Community-level allocation and analysis. Statistical methods described in other publication. Dates of data collection (2001–2004) do not line up with dates of intervention (2003–2005), no post-test data.	Weak
Tyrell (2003) [43]^a^	Weak	Weak	NA	Moderate	Weak	Moderate	No description of who was exposed to the project and who weren’t, nor of possible external influences on outcomes.	Allocation on community and individual level. Evaluation on community, organisational and individual level. No statistical analysis (outcomes as percentages only).	Weak
Guenther (2011) [58]	Strong	Weak	NA	Weak	Weak	N/A	All participants were part of the project; not all participants attended every session; it is likely that the results were influenced by other interventions put on the families.	Individual-level allocation and analysis; statistical analyses (frequencies and *t*-test) were appropriate; analysis performed on actual intervention status.	Weak
Salisbury (1998) [44]^a^	Weak	Moderate	NA	Weak	Weak	Moderate	No description of exposure or consistency; no mention of other interventions influencing outcomes; tested for population growth (which didn’t grow)	Unit of allocation and analysis are on organizational level. No statistical analysis.	Weak
Hunt (2010b) [59]	Weak	Weak	Weak	Weak	Weak	Weak	No description of exposure or consistency, potential influence of other interventions running in the communities at the same time.	Unit of allocation community and organisational level, unit of analysis individual level, no statistical analysis.	Weak
Moran (2003) [45]^a^	Moderate	Moderate	NA	Weak	Moderate	Weak	92 % of participants reported awareness of town plan. Outcomes may be influenced by the cycle of optimism and pessimism.	Unit of allocation is community level, analysis is done on community and individual level. No statistical analysis.	Weak
McCalman (2005) [54]	Moderate	Moderate	NA	NA	Weak	Weak	All evaluation participants were exposed to intervention; consistency was not measured; outcomes likely influenced by other factors.	Project was allocated at organizational level, data were collected on community level, cannot be sure whether changes at community level are caused by changes at the organizational level. No statistical analysis	Weak
Quantitative only studies (*n* = 2)									
Jarvie (2008) [46]^a^	Weak	Weak	Weak	Weak	Weak	Strong	One community withdraw temporarily, others stayed. There’s a chance that outcomes are influenced by other developments going on at the same time	No statistical analysis, intervention offered at community level, data gathered at population level.	Weak
Shannon (2001) [47]^a^	Weak	Moderate	N/A	Weak	Weak	Moderate	No description of exposure to intervention or consistency in delivery. Outcomes may be influenced by other factors	Community-level allocation and analysis. Appropriate statistical analysis.	Weak

*Note:* Appraised using the Dictionary for Effective Public Health Practice Project Quality Assessment tool [38]

^a^ Published in peer-reviewed literature

One study (10 %) described withdrawals and drop outs at the community level [46]. One study (10 %) described withdrawals and drop outs at the participant level [53]. Drop outs at the participant level were not applicable for the six studies (60 %) that used either routinely collected data or a one-off survey [42–44, 47, 54, 58]. Two studies (20 %) did not report drop outs [45, 59]. No study described the fidelity of the project. Three studies (30 %) reported on the exposure of participants to the project [45, 54, 58]. Inferential statistical analyses were reported by four studies (40 %) [42, 47, 53, 58].

### Outcomes

A summary of the aims and key outcomes for each study is provided in Table 6. All studies using qualitative methods concluded that community members reported positive project impacts for their community. Two studies (7 %) reported quantitative outcomes that were statistically significant: a reduction in injuries [47] and a reduction in cannabis use among females aged 13–36 and males aged over 16 years [42].

**Table 6:** Aims and outcomes of studies evaluating Indigenous community development project (*n* = 31)

First author (year)	Project aim	Outcomes of study
Gauld et al. (2011) [40]	Developing culturally relevant rehabilitation service for adults with acquired brain injury.	Experienced increase in knowledge about and access to services for people with acquired brain injuries.
Green et al. (2009) [67]	Community empowerment through arts and cultural practice	Social issues addressed; Non-indigenous staff reported improved understanding Indigenous issues; community members acquiring new skills; experienced increase in supportive relationships and friendships
McMurray (2012) [49]	Increase self-determination of women of the community	Increased networking; improved community governance; increased livelihood opportunities.
Parker et al. (2006) [48]	Introduce Indigenous games in schools to increase physical activity	Process evaluation: most people were satisfied with forum, workshops and activities; project is transferred to other communities.
Murphy et al. (2004) [41]	Enable indigenous people to identify positively with their culture	Youth experienced acquiring wide range of skills; development of pride and connectedness to community and culture; improved self-esteem.
Hunt (2010a) [59]	Encourage healing and harmony for and between (non) Aboriginal people. (Partnership Oxfam and Yorgum)	Family issues were addressed; improved parenting skills; less stress; behavioural change; empowerment.
CLC (2012a) [60]	Increase community understanding and control of usage of mine royalties to facilitate community development	Increased community understanding and perceived and actual community control; increase perceived benefits of project and increase in projects that support the whole community.
CLC (2012b) [51]	Use aboriginal royalties to support education and training initiatives	Perceived improvements in school performance and increase youth employment; growing capacity and ability of committee. Observed increase in school attendance because of school excursions.
CLC (2012c) [61]	Improve quality of dialysis service	Service now strongly contributes to health and wellbeing of family, patients and community
CLC (2012d) [62]	Develop community initiatives and plans for commercial enterprises	Good relationships are built with stakeholders; increased perceived community control; creating activities for community.
CLC (2012e) [63]	Establishing community development to achieve benefits from income from national parks	Commitment to projects that increase community benefits; planning of projects emerged.
Taylor (2005a) [56]	Build capacity of Indigenous Health Worker(IHW) to address childhood asthma and educate community about asthma.	Increased skill transfer and development of IHW; increased confidence in administering own asthma medication; improved relationships doctors and IHWs
Taylor (2005b) [64]	Revitalizing cultural knowledge through traditional games to improve health and build capacity.	Youth experienced increased confidence. Revitalized cultural pride. Indigenous and non-Indigenous people drawn together; empowering.
Ramsay (2005a) [57]	Increase awareness of nutritional need of children and improve early childhood health.	Observed increased awareness nutritional needs, decrease failure to thrive kids and increase in fruits and vegetables in store. Increase confidence in buying healthy food; increase of healthier kids in community. Establishment of community garden.
Ramsay (2005b) [68]	Developing and publishing literacy resources to improve literacy	Publishing and increased use of picture dictionary as effective tool to teach English as a second language.
Burchill (2005) [65]	Revitalizing cultural knowledge through multimedia databases and developing computer skills.	Observed improvement of computer and literacy skills; increase in self-pride and pleasure; generations are drawn together.
Higgins (2005) [52]	Empower Indigenous youth and strengthen links with their culture	Experienced increase in job offers, improved wellbeing of youth. Reported increase in youth entering higher education; decreased expulsions.
Bromfield (2005) [55]	Develop confidence, self-esteem and pride in Indigenous history,	Emerging of real career pathways; observed change in children’s confidence and behaviour.
Ramsay (2005c) [66]	Identify and assist emerging youth community leaders	Youth getting more active in community; youth staying in school longer; observed increase in youth taking employment opportunities
Tsey (2003) [69]	Improve physical, mental, emotional and spiritual wellbeing of individuals and families.	Experienced improvements in parenting skills and confidence; improved student behaviour.
Tsey et al. (2004); [39]	Restore men’s rightful place in the community	Progress towards goal; increase in self-awareness and confidence; taking more responsibility in family life; no improvement in addiction problems.
Smith (2004) [53]	Improving child growth and increasing community involvement.	Increased understanding between community and staff of health service; increased community action; no improvements of child growth.
Lee et al. (2008) [42]	Address youth substance misuse and crime and develop youth activities	No changes in school attendance (2003: 55.9 %; 2005: 51.3 %), or youth apprehension (2003: 68; 2005: 75); decline in cannabis use (2001: 80 %; 2004: 74 %, *p* = .003), statistically significant for females (13–36 years, *p* = .008) and older males (>16 years, *p* = .007).
Tyrrell et al. (2003) [43]	Improve knowledge about and management of diabetes	Increase in visits to health professionals; improved adherence to diabetes management protocol; 65 % decrease in sugar purchases; increase in fruit (81 %) and vegetable (11 %) purchases; no change in biochemical control.
Guenther (2011) [58]	Strengthen and empower families to help children succeed in life	Non-significant increase in school attendance (48.4 to 53 %; *p* > .1); improvements in family environment; no improvements in parental involvement in education; children show more respect towards teachers and other children.
Salisbury (1998) [44]	Improve development and delivery of Aboriginal and Torres Strait Island Mental Health service	Increase in utilization of the service (1994: 73 people; 1997: 770 people using service).
Hunt (2010b) [59]	Build financial capacity in Aboriginal communities	Increased knowledge, confidence and understanding of financial and money management; increase in employment and re-engagement with education.
Moran (2003/2004) [45]	Establishing healthy and sustainable living environment	New healthy settlement was creating; satisfaction of tenants with new buildings; dissatisfaction with level of involvement
McCalman (2005) [54]	Restore men’s rightful place in the community	Reduced injury and suicide rate in community; increase in self-esteem and confidence; increase in seeking help instead of going to drugs.
Jarvie (2008) [46]	Improve relationship between communities and government and build community capacity.	Reduction Indigenous students in lowest literacy bands (2005: 16 %; 2006: 6 %); increase in TAFE enrolments (2001: 1480; 2006: 1718); 32 % increase year 11 and 12 completions; 71 % increase of students finishing certificates and 50 % increase in diplomas; 45 % drop alcohol related hospitalisations, 13 % drop in diabetes-related hospitalisations; decrease in thefts (21.6 %) and breaks (15.8 %) from dwellings.
Shannon et al. (2001) [47]	Reduce injuries in the community	Significant reduction in frequency of injuries before (96; SE = 4.8) and after (65; SE = 3.08) start of the project (Student’s t = 5.07, df = 21, *p* < 0.001).

## Discussion

This study systematically reviewed the peer-reviewed and grey literature on community development projects in Australian Indigenous communities. One hundred and twelve relevant and available publications were identified, 31(28 %) studies were evaluations, 21of these evaluation studies (68 %) were published in grey literature. There were no marked differences observed between evaluations published in the grey and peer-reviewed literature in terms of the detailed descriptions of the qualitative methods used, the quality of the quantitative methods or the reported levels of community participation. This high comparability reflects the generally low quality of all the evaluations published in both the peer-review and the grey literature. It would be an asset to the community development field to increase the publication rate of higher-quality evaluation studies in the peer reviewed literature, especially in open access journals, to utilise peer review as a quality assurance mechanism and to optimise the transparency of study results.

### Community participation in community development projects

Community participation was assessed as moderate in most of the studies evaluating Indigenous community development projects (87 %). The wide variation in community participation between projects and project phases, and within project phases, in these Australian Indigenous studies is reflected in the international literature [32, 36]. For half of the studies included in this review, the intent for community participation was clearly described, but the actual level of participation was not reported for at least one of the phases of project development. Documenting the community participation strategies and processes used, including details about how the community was engaged and who in the community participated, would allow the more successful community participation strategies to be identified and replicated in subsequent projects [36].

Although the unique characteristics of each community will lead to variation in their capacity to participate in each phase of a project [36, 71], the extent and nature of community participation can be optimised by careful planning and the utilisation of appropriate frameworks to guide the development, implementation and evaluation of community-based projects. An approach like participatory action research provides practical guidelines to achieve this [72, 73].

### Quality of evaluation methodology

In line with previous research [4–12], the methodological quality of the studies identified in this review are poor, or they are difficult to assess because their methods are inadequately described. It is acknowledged that issues specific to Indigenous community-based research can impact on the research quality, including time needed to engage with the community, difficulties with recruiting enough participants, high staff turn-over at service providers and culturally-specific delays (e.g. ceremonies or celebrations) [14]. Careful and flexible planning is therefore needed in community development projects to address these issues to reduce their impact on the quality of the research. The complex interventions framework, for example, provides one mechanism to carefully plan projects to maintain methodological rigour [74, 75]. The methodological quality of qualitative studies could also be improved by using appropriate analysis methods, multiple coders, and describing the extent of potential bias attributable to the researcher [37].

The methodological rigour of both the qualitative and the quantitative studies could further be improved by using measures with demonstrated reliability and validity: only two studies identified by this review reported that they had used such measures [45, 58]. Using reliable and valid measures increases confidence in the accuracy of the study outcomes [76]. Such measures should be validated specifically for the Indigenous population, because of their holistic concept of health and wellbeing [77]. Existing studies show that it is possible to develop reliable and valid measures that are culturally appropriate and acceptable to Indigenous Australians [78–83], but the lack of measurement studies specifically related to community projects identified in this review (one study, see Fig. 1) clearly indicates that more of this measurement research is urgently needed [80].

Only three studies (10 %) reported on intervention integrity, which includes the level of exposure to the project, and the consistency and frequency with which project components were delivered in practice. Studies evaluating community development projects would be improved by routinely including process measures, to allow an examination of the extent to which outcomes are a consequence of the project components, as opposed to reflecting the extent to which the project components were implemented [74].

Eight studies (26 %) evaluated a community development project using a mixed methods design. Increasing the use of mixed methods is likely to optimally improve the effectiveness of future community-based evaluations because they provide a greater range of relevant data [11, 84]: quantitative analysis can provide rigorous methods to evaluate the effectiveness and costs of projects, while qualitative data can capture community members’ experiences [85] and help identify the project elements that are most acceptable to community members [11].

The critical appraisal also identified a lack of detailed reporting of the methodologies used, especially in relation to the qualitative evaluations. Only 41 % of the qualitative studies reported on their data collection process, for example, and only 24 % reported the data analysis methods that were used. Future Indigenous community development evaluations would benefit from more detailed reporting using established guidelines, such as the COREQ criteria for qualitative research [86] or the guidelines recommended by the Equator Network [87]. In addition to improving reporting standards, using these guidelines in the development, implementation and evaluation phases of community development projects would most likely improve the quality of the interventions and their evaluation [37, 38].

### Outcomes of indigenous community development projects

There is currently insufficient evidence about the impact of community development projects on health and wellbeing outcomes for Indigenous Australians. Although all reviewed studies reported positive outcomes for the communities, they are not methodologically rigorous enough to support clear conclusions about their cost-effectiveness, and no studies have undertaken an economic analysis to weigh the benefits of community development against its costs. This finding is highly consistent with the conclusions of similar reviews of international Indigenous community development studies, where generally positive outcomes are difficult to interpret because of the relatively poor quality of their evaluation designs and reporting [9, 19, 88]. Published results of community-based evaluations with greater methodological quality are required to provide evidence of cost-effective community development projects [9, 74]. Ideally, future studies would use rigorous evaluation designs, reliable, valid and culturally appropriate measures, economic analysis and a complex intervention framework to balance standardisation and tailoring.

### Strengths and limitations

To ensure that qualitative and quantitative study components were assessed against appropriate criteria the Dictionary for Effective Public Health Practice Project Quality Assessment tool [38] was used to assess the methodological quality of quantitative components and an adaptation of the qualitative study appraisal tool, developed by Long and Godfrey [37] was used for the qualitative study components. The methodological quality of the studies and extent of community participation may have been misclassified, however the high level of agreement between blinded coders suggest not. Of the 231 full-text articles sought for detailed review, 40 (17 %) were excluded because the full text version of these papers were unable to be accessed. Excluding these 40 papers is unlikely to have compromised the comprehensiveness of this review for three reasons: 1) they only represent 17 % of the full-text articles; 2) the majority were older studies or reports that were not publically available; and 3) the references lists of identified publications were hand searched and researchers in the field were consulted to identify publications not found by the electronic database search.

## Conclusion

This systematic review identified that levels of community participation fluctuate across community development project phases: moderate in the Diagnosis and Development phases, high in the Implementation phase, but low in the Evaluation phase. It also identified that the methodological quality of studies evaluating Australian Indigenous community development projects is too weak to confidently determine the cost-effectiveness of these projects in improving the health and wellbeing of Indigenous Australians. Studies of greater methodological quality are required to accurately assess the impact of community development projects. Partnerships combining researchers’ expertise and community members’ skills and knowledge have great potential to improve methodological quality and community participation in Indigenous community development projects [9, 11, 89].
